# Role of DCLK1/Hippo pathway in type II alveolar epithelial cells differentiation in acute respiratory distress syndrome

**DOI:** 10.1186/s10020-023-00760-0

**Published:** 2023-11-23

**Authors:** Xiao-Yue Chen, Ching Kao, Syue-Wei Peng, Jer-Hwa Chang, Yueh-Lun Lee, Vincent Laiman, Kian Fan Chung, Pankaj K. Bhavsar, Didik Setyo Heriyanto, Kai-Jen Chuang, Hsiao-Chi Chuang

**Affiliations:** 1 Graduate Institute of Medical Sciences, College of Medicine, Taipei Medical University, Taipei, Taiwan; 2 School of Respiratory Therapy, College of Medicine, Taipei Medical University, 250 Wuxing Street, Taipei, 11031 Taiwan; 3 National Heart and Lung Institute, Imperial College London, London, UK; 4 Division of Pulmonary Medicine, Department of Internal Medicine, Wan Fang Hospital, Taipei Medical University, Taipei, Taiwan; 5 Department of Microbiology and Immunology, School of Medicine, College of Medicine, Taipei Medical University, 250 Wuxing Street, Taipei, 11031 Taiwan; 6 International Ph.D. Program in Medicine, College of Medicine, Taipei Medical University, Taipei, Taiwan; 7 Department of Anatomical Pathology, Faculty of Medicine, Public Health, and Nursing, Universitas Gadjah Mada, Dr. Sardjito Hospital, Yogyakarta, Indonesia; 8 School of Public Health, College of Public Health, Taipei Medical University, Taipei, Taiwan; 9 Department of Public Health, School of Medicine, College of Medicine, Taipei Medical University, Taipei, Taiwan; 10 Division of Pulmonary Medicine, Department of Internal Medicine, Shuang Ho Hospital, Taipei Medical University, New Taipei City, Taiwan; 11 Cell Physiology and Molecular Image Research Center, Wan Fang Hospital, Taipei Medical University, Taipei, Taiwan; 12 Inhalation Toxicology Research Lab (ITRL), School of Respiratory Therapy, College of Medicine, Taipei Medical University, 250 Wuxing Street, Taipei, 110 Taiwan

**Keywords:** Epithelium, Infection, Lung injury, Pneumocytes, Regeneration

## Abstract

**Background:**

Delay in type II alveolar epithelial cell (AECII) regeneration has been linked to higher mortality in patients with acute respiratory distress syndrome (ARDS). However, the interaction between Doublecortin-like kinase 1 (DCLK1) and the Hippo signaling pathway in ARDS-associated AECII differentiation remains unclear. Therefore, the objective of this study was to understand the role of the DCLK1/Hippo pathway in mediating AECII differentiation in ARDS.

**Materials and methods:**

AECII MLE-12 cells were exposed to 0, 0.1, or 1 μg/mL of lipopolysaccharide (LPS) for 6 and 12 h. In the mouse model, C57BL/6JNarl mice were intratracheally (i.t.) injected with 0 (control) or 5 mg/kg LPS and were euthanized for lung collection on days 3 and 7.

**Results:**

We found that LPS induced AECII markers of differentiation by reducing surfactant protein C (SPC) and p53 while increasing T1α (podoplanin) and E-cadherin at 12 h. Concurrently, nuclear YAP dynamic regulation and increased TAZ levels were observed in LPS-exposed AECII within 12 h. Inhibition of YAP consistently decreased cell levels of SPC, claudin 4 (CLDN-4), galectin 3 (LGALS-3), and p53 while increasing transepithelial electrical resistance (TEER) at 6 h. Furthermore, DCLK1 expression was reduced in isolated human AECII of ARDS, consistent with the results in LPS-exposed AECII at 6 h and mouse SPC-positive (SPC^+^) cells after 3-day LPS exposure. We observed that downregulated DCLK1 increased p-YAP/YAP, while DCLK1 overexpression slightly reduced p-YAP/YAP, indicating an association between DCLK1 and Hippo-YAP pathway.

**Conclusions:**

We conclude that DCLK1-mediated Hippo signaling components of YAP/TAZ regulated markers of AECII-to-AECI differentiation in an LPS-induced ARDS model.

**Graphical Abstract:**

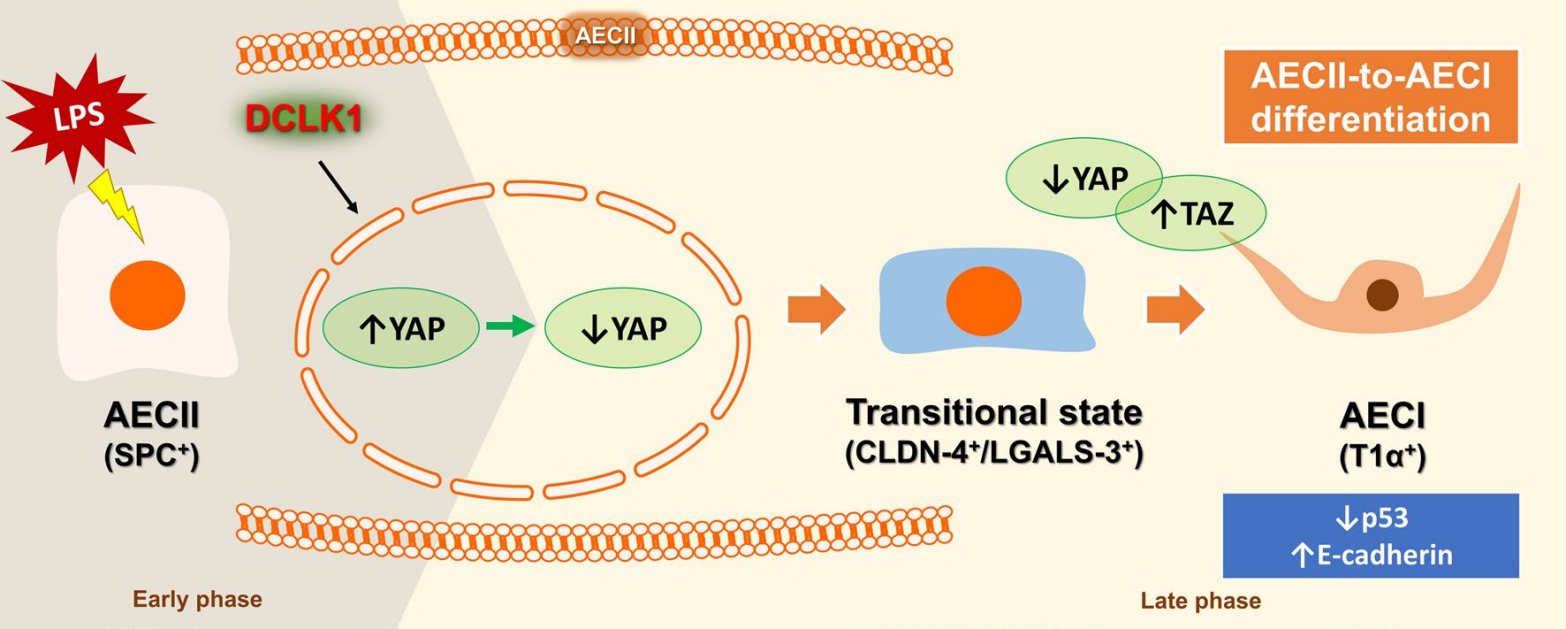

## Highlights

1. LPS activated AECII-to-AECI markers of differentiation via the Hippo signaling pathway.
2. DCLK1/YAP was dynamically regulated in AECII of LPS-induced ARDS.
3. DCLK1 had an association with YAP to increase cell stemness in AECII.
4. DCLK1/Hippo pathway could be a potential therapeutic target in patients with ARDS.

## Introduction

Acute respiratory distress syndrome (ARDS) causes heterogeneous pulmonary destruction and deoxygenation (Auriemma et al. 2020), which can result in an increased mortality rate of 30–40% in patients with ARDS due to a resulting inability to repair the lungs (Bime et al. 2019). Unresolved lung injury could also progress to pulmonary fibrosis with chronic hypoxemia, ultimately reducing the 6-min walking distance in ARDS patients (Gao et al. 2021). In the pathogenesis of ARDS, gram-negative bacteria are the predominant cause (Bos and Ware 2022; Umbrello et al. 2016), with bacterial lipopolysaccharide (LPS) interacting with Toll-like receptor 4 to induce the production of interleukin (IL)-1β and tumor necrosis factor (TNF)-α (Domscheit et al. 2020; Zhou et al. 2018). These proinflammatory cytokines activate alveolar macrophages and neutrophils, leading to pulmonary capillary leakage and alveolar epithelial integrity loss by reducing transepithelial electrical resistance (TEER) in the human alveolar epithelium (Metz et al. 2020) during the early phase of ARDS (Potey et al. 2019; Zhou et al. 2018). During the late phase of ARDS, Type II alveolar epithelial cells (AECII) become resistant to lung injury and differentiate to repair the damaged Type I alveolar epithelial cells (AECI) due to their high progenitor capacity (Kasper and Barth 2017; Zeng et al. 2016). It has been observed that the increased TEER was associated with the restoration of barrier integrity during AECII-to-AECI differentiation (Ishii et al. 2021). Additionally, it is important to note that surfactant protein C (SPC) serves as a specific marker for AECII detection (LaCanna et al. 2019). Furthermore, during the differentiation process, AECII transiently expresses claudin 4 (CLDN-4) and galectin 3 (LGALS-3), transitioning into the pre-alveolar type-1 transitional cell state (PATS), which eventually differentiates into AECI (Kobayashi et al. 2020). Also, T1α (podoplanin) levels can be employed as a specific marker expressed on AECI (LaCanna et al. 2019; Ramirez et al. 2003). However, persistent inflammation can impede the differentiating function of AECII. Chronic elevation of IL-1β, for example, interferes with AECII-to-AECI differentiation after bleomycin-induced murine lung injury (Choi et al. 2020). Moreover, interferon (IFN)-γ causes immune-mediated lung damage and delays resolution of ARDS (Mock et al. 2020). Dysfunction of AECII can lead to severe ARDS and pulmonary fibrosis (Auriemma et al. 2020; Kobayashi et al. 2020). Therefore, understanding the repair mechanism of AECII differentiation is crucial in order to improve the poor outcomes of ARDS patients.

The Hippo pathway plays a critical role in embryological lung development and the maintenance of AECII survival (Hu et al. 2019; Lange et al. 2015). Yes-associated protein (YAP) and its transcriptional co-activator with PDZ-binding motif (TAZ), the essential proteins regulated in the Hippo pathway, and the dephosphorylation of cytoplasmic YAP/TAZ induce their translocation into the nucleus, with activation of downstream signals for cell development (Boopathy and Hong 2019). We have previously demonstrated that umbilical cord-derived stem cells dynamically regulated YAP to mediate AECII-to-AECI differentiation with an acceleration of inflammatory resolution in mice with LPS-induced ARDS (Chen et al. 2023). This suggests that YAP may be involved in the repair process of ARDS. Similarly, TAZ was found to cause AECII-to-AECI differentiation after bleomycin-induced lung injury in mice (Sun et al. 2019). However, overexpression of YAP/TAZ has been shown to enhance the epithelial-mesenchymal transition in cancer (Chen et al. 2016; Yuan et al. 2016), and increased epithelial YAP activity has been reported in the lungs of patients with idiopathic pulmonary fibrosis (IPF) (Gokey et al. 2018). Therefore, it is crucial to fine-tune the regulation of the Hippo pathway because any dysregulation can lead to pathological lung reconstruction (Gokey et al. 2021). However, the pathophysiological regulation of the Hippo pathway in AECII differentiation remains unclear, and the upstream mediators regulating the Hippo pathway are not known in ARDS.

Doublecortin-like kinase 1 (DCLK1) has been reported to enhance cell regeneration and proliferation (Chandrakesan et al. 2017; Westphalen et al. 2017) and is highly expressed in cancer, progenitor cells, and type 2 macrophages (Panneerselvam et al. 2020; Undi et al. 2022; Westphalen et al. 2016). Increased inflammatory signals and tissue damage activate DCLK1 expression (Nguyen et al. 2016; Undi et al. 2022). Furthermore, the upregulated DCLK1 gene was found in the lungs of patients with IPF (Higo et al. 2022). DCLK1 has been shown to elongate microtubules for cell growth, but excessive elongation can increase cancer cell development (Chhetri et al. 2022). Another potential role of DCLK1 is associated with lung regeneration, as previous studies have shown an increased expression of DCLK1-positive cells during dysplastic lung repair in influenza and bleomycin-induced lung injury (Barr et al. 2022; Rane et al. 2019). However, the expression of DCLK1 in AECII after lung injury has not been fully addressed, and the interaction between DCLK1 and the Hippo pathway in ARDS is unclear. A greater understanding of the underlying process of alveolar regeneration could provide important information for developing new drugs to treat ARDS patients. Therefore, this study aims to explore the roles of the DCLK1-modulated Hippo pathway in AECII-to-AECI differentiation after LPS-induced ARDS.

## Materials and methods

### Gene Expression Omnibus (GEO) dataset

RNA-sequencing data from human lung tissue was obtained from the GEO dataset (Fang et al. 2015). Briefly, human AECII were isolated and collected from 5 healthy male subjects. AECII was exposed to a cytokine mixture with interleukin (IL)-1β, tumor necrosis factor (TNF)-α, and interferon (IFN)-γ at 50 ng/mL to establish an ARDS in vitro model. DCLK1 expression in control and ARDS groups was collected from the dataset.

### LPS-exposed AECII

Mouse AECII MLE-12 cells were obtained from ATCC (American Type Culture Collection, Manassas, Virginia, USA). These cells display gene expression profiles that include SPC, a characteristic specific to AECII (LaCanna et al. 2019). Furthermore, previous studies have commonly utilized MLE-12 cells in vitro to model AECII behavior, thereby facilitating investigations into the underlying mechanisms of AECII related to pulmonary diseases (Bueno et al. 2023; Li et al. 2019; Lu et al. 2023). MLE-12 cells were cultured in Dulbecco’s modified Eagle’s medium (DMEM)/Hams F-12 50/50 mix (Corning, Corning, NY, USA) at 37 °C under 5% CO_2_. The medium contained 10% fetal bovine serum (Corning), 5 μg/mL insulin (Sigma-Aldrich, St. Louis, USA), 10 μg/mL transferrin (Sigma-Aldrich), 30 nM sodium selenite (Sigma-Aldrich), 10 nM hydrocortisone (Sigma-Aldrich), 10 nM β-estradiol (Sigma-Aldrich), and 1% penicillin/streptomycin (Corning). Cells were treated with LPS (*Escherichia coli* O111:B4; Sigma-Aldrich) at 0 (control), 0.1 (LPS/L), or 1 μg/mL (LPS/H) for 6 and 12 h. The time points of 6 and 12 h were chosen to respectively delineate the early and late phases of infection-induced AECII cell injury.

### Transfection of YAP or DCLK1 in AECII

The MLE-12 cells were transfected with MISSION® esiRNA (Sigma-Aldrich) to knockdown the expression of YAP1 or DCLK1, according to the manufacturer’s instructions. The cells were transfected with 50 nM of small interfering RNAs (siRNA) targeting YAP (Si-YAP), DCLK1 (Si-DCLK1), or negative control (Si-Control) (GAUCAUACGUGCGAUCAGA/UCUGAUCGCACGUAUGAUC) using Lipofectamine 3000 transfection reagent (Invitrogen, Carlsbad, CA, USA) for 48 h.

### DCLK1 overexpressed in AECII

DCLK1 overexpression was conducted by the DCLK1 (Myc-DDK-tagged) open reading frame (ORF) clone (OriGene Technologies, Rockville, MD, USA). The plasmid was subsequently amplified by BIOTOOLS, New Taipei City, Taiwan. The MLE-12 cells were transfected with DCLK1 cDNA (OE-DCLK1) or the negative control with empty vector (OE-Control) (GACGGATCGGGAGATCTCCCGATCCCCTATGGTGCACTCCAGTACAATCTGCTCTGATG) by Lipofectamine 3000 and p3000 transfection reagents (Invitrogen) at a final concentration of 5 μg/μL for 24 h.

### Cytoplasmic and nuclear protein extraction

The cytoplasmic and nuclear proteins of YAP/TAZ and DCLK1 in MLE-12 cells were extracted using a cytoplasmic and nuclear protein extraction kit (BIOTOOLS, New Taipei City, Taiwan) according to the manufacturer’s instructions. Briefly, cytoplasmic extraction reagents (BIOTOOLS) in the kit were added to cell pellets and incubated on ice. Cells were centrifuged at 16,000×*g* for 5 min to collect supernatants. After cytoplasmic protein extraction, a nuclear extraction reagent (BIOTOOLS) was added to the nuclei-containing insoluble cell debris, followed by centrifugation at 16,000×*g* for 5 min. Additionally, whole cell lysates were obtained by subjecting the cells to lysis using cell lysis reagent (Sigma-Aldrich, St. Louis, MO, USA) supplemented with 5 μL of protease inhibitor (G-Biosciences, St. Louis, MO, USA) and 5 μL of ethylenediaminetetraacetic acid (EDTA; G-Biosciences) for detecting E-cadherin, SPC, CLDN-4, LGALS-3, T1α, and p53 levels.

### Western blot

Protein concentrations of cell lysates were determined using a bicinchoninic acid (BCA) protein assay (Bio-Rad, Hercules, CA, USA). Cell protein extracts were separated on SDS–polyacrylamide gels and transferred to polyvinylidene difluoride membranes (Millipore, Darmstadt, Germany). The protein expressions in the same replicates were blotted on separate parallel gels and membranes. Membranes were incubated overnight at 4 °C probed with primary antibodies of E-cadherin (1:1000) (Abcam plc., Cambridge, UK), surfactant protein C (SPC; 1:5000) (Signalway Antibody, Greenbelt, MD, USA), claudin 4 (CLDN-4; 1:200) (Santa Cruz Biotechnology, Dallas, TX, USA), galectin 3 (LGALS-3; 1:1000) (Cell Signaling, Danvers, MA, USA), podoplanin (T1α; 1:1000) (Abcam), p53 (1:1000) (Proteintech, Rosemont, IL, USA), phosphorylated form (p-) of YAP (p-YAP; 1:1000) (Abcam), YAP (1:1000) (Proteintech), p-TAZ (1:1000) (Cell Signaling), TAZ (1:1000) (Cell Signaling), DCLK1 (1:1000) (GeneTex, Irvine, CA, USA), β-actin (1:1000) (Proteintech), and Lamine A+C (1:500) (GeneTex). The mouse and rabbit secondary antibodies (1:5000) (Jackson Immunoresearch, West Grove, PA, USA) with enhanced chemiluminescence (PerkinElmer, Waltham, MA, USA) were used. Images were taken with the ChemiDoc™ MP Imaging System (Bio-Rad). All proteins in the images were semi-quantified using Image-Pro version 4 (Media Cybernetics, Rockville, MD, USA). The expression of cytoplasmic proteins was normalized by the internal control (β-actin) within the same repetition, while the nuclear levels of YAP, TAZ, and DCLK1 were normalized by their nuclear Lamin A+C expression.

### Transepithelial electrical resistance (TEER)

MLE-12 cultured on 0.4-μm transwell (SPL®, Gyeonggi-do, Korea) were treated with 0.16 μg/mL or 0.33 μg/mL of YAP inhibitor verteporfin (MedChemExpress LLC, New Jersey, USA) for 2, 4, and 6 h. The concentrated verteporfin was diluted in the pure MLE-12 cell medium. The cells treated with the medium alone served as control. TEER was measured between the inner and outer chambers of the transwell by an Epithelial Volt/Ohm Meter (World Precision Instruments, Sarasota, FL, USA).

### LPS-induced ARDS mouse model

Male C57BL/6JNarl mice (8 weeks old, 20–25 g), obtained from the National Laboratory Animal Center (Taipei, Taiwan), were intratracheally instilled (*i.t.*) with 0 (control) and 5 mg/kg LPS (*Escherichia coli* O111:B4; Sigma-Aldrich) to establish ARDS model. Mice were euthanized on days 3 and 7 after LPS administration. Days 3 and 7 corresponded to the early and late phases of ARDS, as previously demonstrated in our study (Chen et al. 2023). Lungs were collected and preserved in 10% neutral buffered formalin.

### Immunofluorescence (IF)

Paraffin-embedded lung tissues were sectioned and permeabilized using 0.25% Triton X-100 (BIONOVAS Biotechnology, Ontario, Toronto, Canada). The 5% of bovine serum albumin (BSA) was used to block lung tissues, followed by incubating with primary antibodies of SPC (1:200) (Abcam) and DCLK1 (1:500) (Abcam). The rabbit and goat secondary antibodies (1:200) (Abcam) were used to conjugate with the primary antibodies. DCLK1 was detected using Alexa Fluor 488 (Abcam), while SPC was detected using Alexa Fluor 555 (Abcam). Nuclear counterstaining was performed with 4ʹ,6-diamidino-2-phenylindole (DAPI) (Abcam). Fluorescent images were captured using a confocal fluorescence microscope (TCS SP5, Leica, UK). The mean fluorescence intensity of DCLK1 (DCLK1^+^) on SPC positive (SPC^+^) cells was quantified using Image J software (National Institutes of Health, Bethesda, MD, USA) based on a previous report (Shihan et al. 2021).

### Statistical analysis

Data are presented as the mean ± standard deviation (SD). Student’s *t*-test was used to compare two continuous variables. For comparisons among multiple variables, one-way analysis of variance (ANOVA) with Dunnett’s post hoc test was used. Statistical analyses were performed using GraphPad vers. 7 (San Diego, CA, USA). p-value < 0.05 was considered as statistical significance.

## Results

### AECII-to-AECI differentiation by LPS

**Fig. 1:**
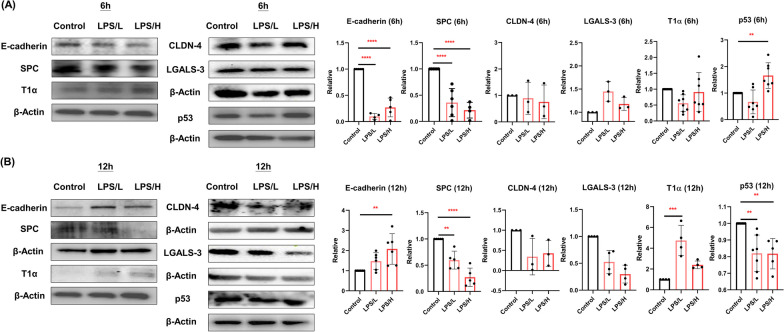
Lipopolysaccharide (LPS) induced type II alveolar epithelial cells (AECII)-to-type I alveolar epithelial cells (AECI) markers of differentiation. Semi-quantification of E-cadherin, surfactant protein C (SPC), claudin 4 (CLDN-4), galectin 3 (LGALS-3), podoplanin (T1α), and p53 in MLE-12 cells by western blot after 0 μg/mL (control), 0.1 μg/mL (LPS/L), or 1 μg/mL (LPS/H) of LPS exposure for **A** 6 h and **B** 12 h. All data are presented as mean ± SD with a minimum of three independent experiments. An ANOVA with Dunnett’s post hoc test was used. ** *p* < 0.01, *** *p* < 0.001, **** *p* < 0.0001

Figure 1 A and B show the effect of LPS exposure on AECII-to-AECI differentiation for 6 and 12 h. We found a significant decrease in E-cadherin and SPC but an increase in p53 expression by LPS after 6 h exposure to MLE-12 cells (*p* < 0.05). Next, we demonstrated that LPS significantly decreased SPC and p53; however, levels of E-cadherin (by LPS/H) and T1α (by LPS/L) were significantly increased by LPS after 12 h exposure (*p* < 0.05). There was no significant difference in CLDN-4 and LGALS-3 expression by LPS for 6 or 12 h.

### *AECII-to-AECI differentiation through Hippo pathway after LPS* exposure

**Fig. 2:**
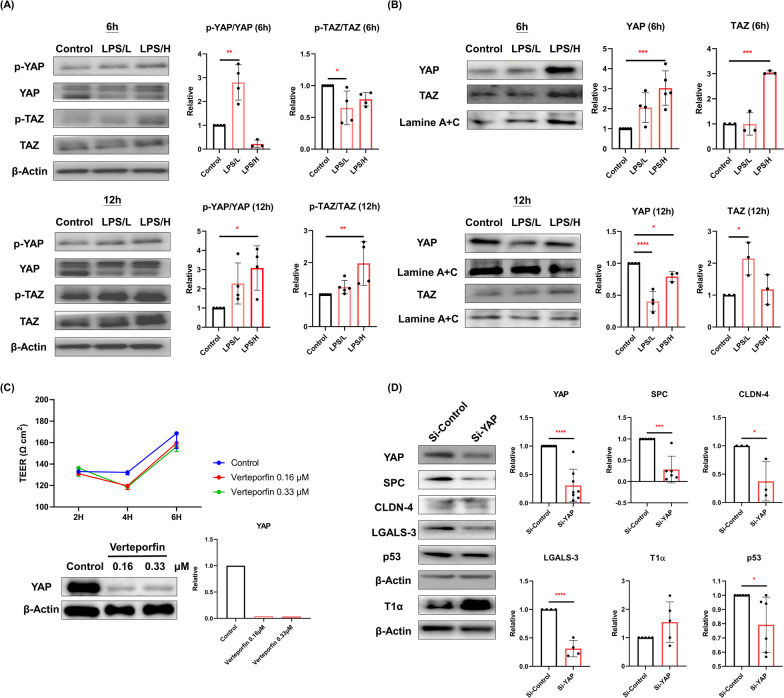
Type II alveolar epithelial cells (AECII) differentiated through the Hippo pathway after exposure to lipopolysaccharide (LPS). **A** Cytoplasmic levels of phosphorylated (p-) YAP-to-YAP (p-YAP/YAP) and p-TAZ/TAZ ratio (**B**) and the nuclear YAP and TAZ expressed in MLE-12 cells by western blot after exposed to 0 μg/mL (control), 0.1 μg/mL (LPS/L), or 1 μg/mL (LPS/H) of LPS for 6 h and 12 h. **C** The epithelial permeability of MLE-12 cells was assessed by transepithelial electrical resistance (TEER) while treated with verteporfin (0 μM, 0.16 μM, or 0.33 μM) at 2 h, 4 h, and 6 h. **D** Protein expression of YAP, surfactant protein C (SPC), claudin 4 (CLDN-4), galectin 3 (LGALS-3), podoplanin (T1α), and p53 in MLE-12 cells after transfected with small interfering RNA (Si-) of YAP (Si-YAP) or Si-Control at 50 nM for 48 h. All data are presented as mean ± SD with a minimum of three independent experiments in **A**, **B**, **D** and one experiment in **C**. An ANOVA with Dunnett’s post hoc test was employed for (**A**) and (**B**), while (**D**) was analyzed using a Student’s t-test. * *p* < 0.05, ** *p* < 0.01, *** *p* < 0.001, **** *p* < 0.0001

As shown in Fig. 2A, we found that the cytoplasmic p-YAP/YAP ratio in MLE-12 cells was significantly increased at 6 and 12 h by LPS/L and LPS/H (*p* < 0.05), respectively. There was a significant decrease in the cytoplasmic p-TAZ/TAZ ratio by LPS/L at 6 h and the subsequent increase in the p-TAZ/TAZ ratio at 12 h after LPS/H exposure (*p* < 0.05). At the nuclear level, we found YAP and TAZ expression were significantly increased at 6 h (*p* < 0.05), whereas a decrease in YAP and an increase in TAZ were observed at 12 h by LPS (*p* < 0.05; Fig. 2B). To assess the barrier integrity during AECII-to-AECI differentiation, we measured the TEER in MLE-12 cells after inhibiting YAP with verteporfin. Decreased epithelial resistance (TEER) at 4 h but increased at 6 h in MLE-12 cells were observed by YAP inhibition (Fig. 2C). Levels of YAP, SPC, CLDN-4, LGALS-3, and p53 were significantly decreased after YAP knockdown (*p* < 0.05; Fig. 2D).

### DCLK1 regulation on AECII of ARDS

**Fig. 3:**
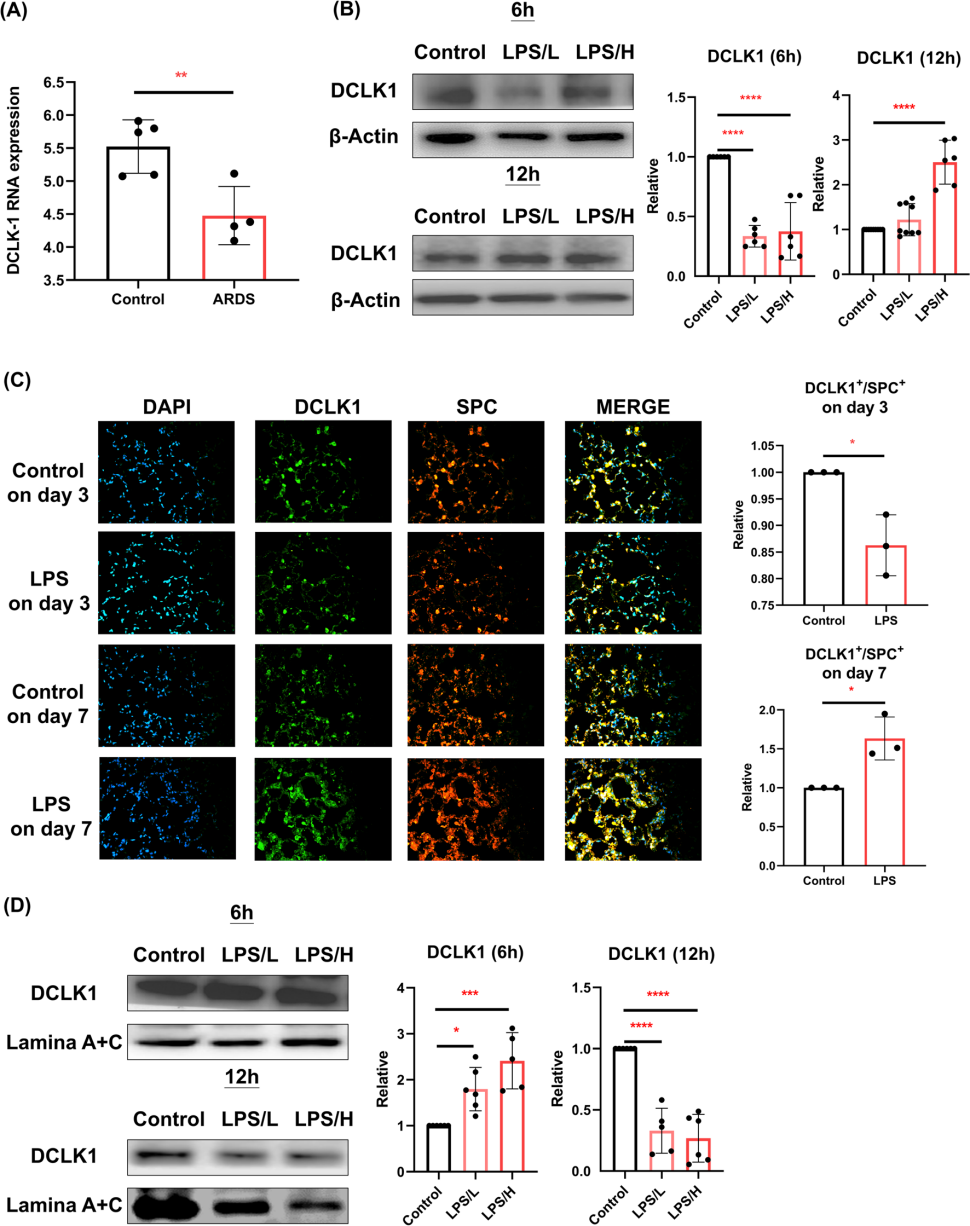
Doublecortin-like kinase 1 (DCLK1) regulation in type II alveolar epithelial cells (AECII) with acute respiratory distress syndrome (ARDS). **A** The DCLK1 mRNA expression in human AECII of ARDS was collected from the Gene Expression Omnibus (GEO) dataset. **B** Western blot showed the cytoplasmic DCLK1 expression in MLE-12 cells after 0 μg/mL (control), 0.1 μg/mL (LPS/L), or 1 μg/mL (LPS/H) LPS exposure for 6 h and 12 h. **C** Immunofluorescent staining of DCLK1 expression (DCLK1^+^) on SPC-positive (SPC^+^) cells in ARDS mouse lungs on days 3 and 7 after exposure to LPS. **D** The nuclear levels of DCLK1 were determined in LPS-exposed MLE-12 cells (0 μg/mL, 0.1 μg/mL, or 1 μg/mL) by Western blot at 6 h and 12 h. All data are presented as mean ± SD with a minimum of four independent experiments in **A**, **B**, and **D** and three experiments in **C**. A Student’s t-test was utilized for (**A**) and (**C**), whereas an ANOVA with Dunnett’s post hoc test was employed for (**B**) and (**D**). * *p* < 0.05, ** *p* < 0.01, *** *p* < 0.001, **** *p* < 0.0001

We observed a significant decrease in the RNA level of DCLK1 on AECII of ARDS (by the GEO dataset) (*p* < 0.05; Fig. 3A). In the LPS-exposed AECII cell model, we found that DCLK1 was significantly decreased in MLE-12 cells at 6 h by LPS, whereas DCLK1 was increased at 12 h after LPS/H exposure (*p* < 0.05; Fig. 3B). The LPS-induced ARDS mouse model consistently showed a significant decrease in DCLK1 expression with SPC^+^ cells on day 3 and an increase in DCLK1 with SPC^+^ cells on day 7 (*p* < 0.05; Fig. 3C). On the contrary, the nuclear level of DCLK1 on MLE-12 cells showed that LPS significantly increased DCLK1 expression at 6 h and reduced DCLK1 expression at 12 h (*p* < 0.05; Fig. 3D).

### DCLK1 modulated AECII-to-AECI differentiation by Hippo pathway

**Fig. 4:**
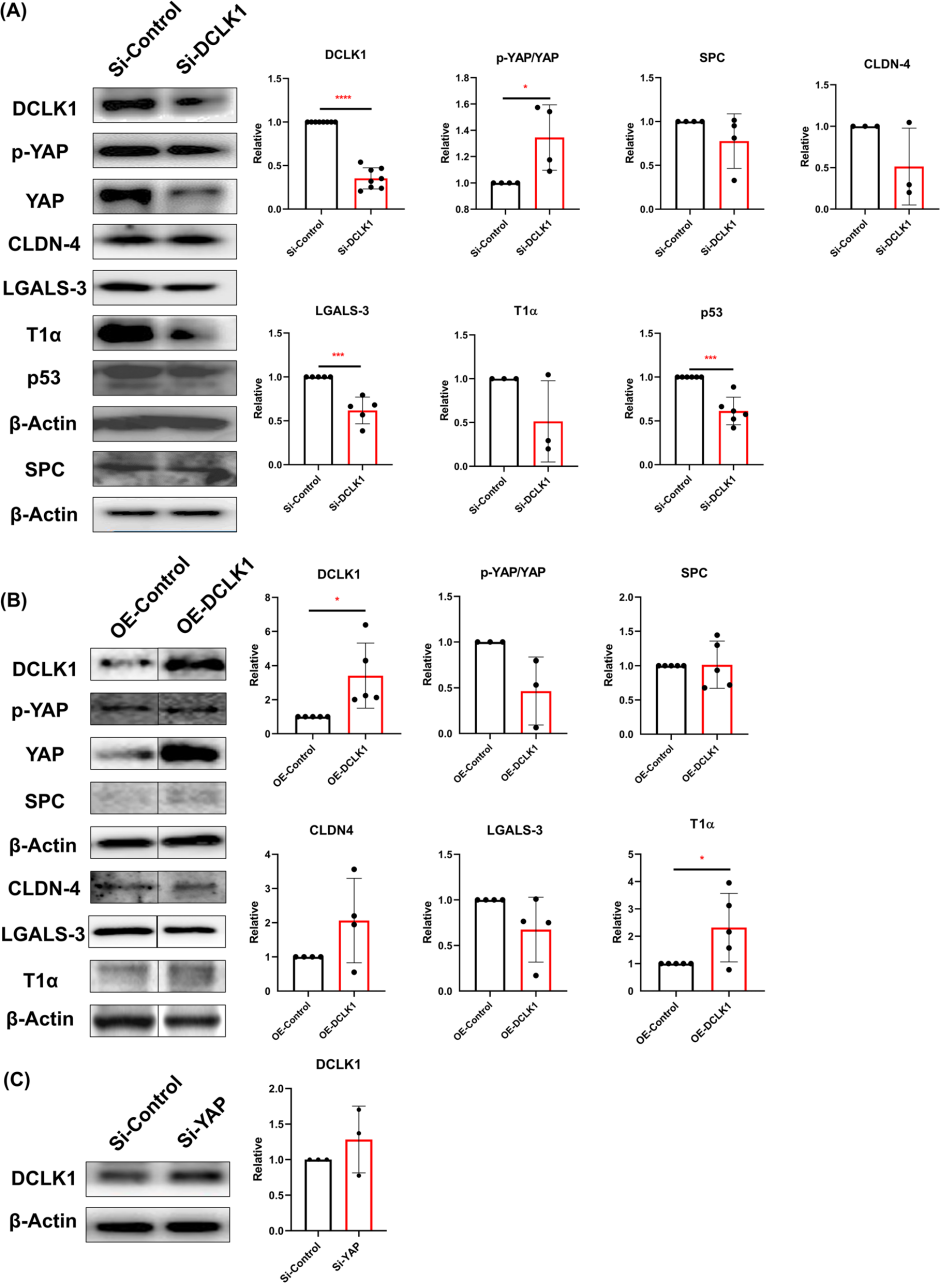
Doublecortin-like kinase 1 (DCLK1) involved in type II alveolar epithelial cells (AECII)-to-type I alveolar epithelial cells (AECI) differentiation via the Hippo pathway. **A** Protein levels of DCLK1, phosphorylated (p-) YAP-to-YAP (p-YAP/YAP) ratio, surfactant protein C (SPC), claudin 4 (CLDN-4), galectin 3 (LGALS-3), podoplanin (T1α), and p53 in MLE-12 cells by 50 nM small interfering RNA (Si-) of DCLK1 (Si-DCLK1) or Si-Control for 48 h. **B** DCLK1, p-YAP/YAP, SPC, CLDN-4, LGALS-3, and T1α were semi-quantified by western blot in MLE-12 cells after DCLK1 overexpression (OE-DCLK1). **C** Expression of DCLK1 in MLE-12 cells after transfection with 50 nM of Si-YAP or Si-Control for 48 h. All data are presented as mean ± SD with a minimum of three independent experiments. A Student’s t-test was applied to analyze the results. * *p* < 0.05, *** *p* < 0.001, **** *p* < 0.0001

We observed that the DCLK1, LGALS-3, and p53 significantly decreased, whereas the p-YAP/YAP ratio significantly increased after the DCLK1 knockdown on MLE-12 cells (*p* < 0.05; Fig. 4A). On the other hand, the overexpression of DCLK1 in MLE-12 cells led to a significant increase in both DCLK1 and T1α expression while showing a trend toward decreasing the p-YAP/YAP ratio (Fig. 4B). Furthermore, we did not observe a significant difference in the level of DCLK1 after YAP knockdown on MLE-12 cells (Fig. 4C).

## Discussion

We have demonstrated that LPS-induced differentiation markers transitioned from AECII into AECI by regulating the Hippo pathway. These results were observed from the alteration in the nuclear levels of YAP/TAZ in LPS-exposed AECII during cell differentiation. The significance of this work is that we observed a shift from reduced cytoplasmic DCLK1 levels during the early phase of ARDS to increased levels in the late phase. These observations suggest dynamic regulation of DCLK1 in AECII during different phases of ARDS, indicating that DCLK1 may have an association with the Hippo pathway to regulate AECII differentiation. We elucidate the underlying mechanisms of alveolar regeneration and differentiation of AECII into AECI by DCLK1/YAP/TAZ pathways, which could aid in the development of therapies that promote tissue repair and regeneration in the lungs of ARDS patients.

We initially investigated the differentiation markers of AECII into AECI following exposure to LPS. We found that at 6 h, LPS caused a decrease in SPC but an increase in p53. The loss of SPC in alveoli due to dysfunctional AECII has been previously reported in gram-negative bacteria-induced ARDS (Wu et al. 2021). It is likely that bacteria target AECII, leading to DNA damage in the cells (Augusto et al. 2003; D'Agnillo et al. 2021), which activates the check-point inhibitor, p53, causing cell cycle arrest (Williams and Schumacher 2016) and initiating a self-repairing process (Liu et al. 2009; Uddin et al. 2020). This process temporarily ceases SPC production in AECII (Glasser et al. 2013). Moreover, bacteria-infected AECII impedes cell differentiation, which was observed in our model of LPS-exposed AECII at 6 h, as evidenced by a reduction in E-cadherin. Interestingly, AECII progenitor cells have a higher self-repairing capacity (Desai et al. 2014). Previous studies showed that AECII exhibit increased self-renewal after ablation in mice (Barkauskas et al. 2013) and can be activated to differentiate into AECI after bacterial pneumonia (Liu et al. 2015), further supporting our observations of increasing T1α and E-cadherin in AECII after 12 h of LPS exposure. This suggests the restoration of differentiating function in AECII. The subsequent reduction of p53 in our cell model at 12 h further indicates the end of cell cycle arrest for self-repairing in LPS-injured AECII. Together, our findings suggest that LPS induces markers of AECII differentiation into AECI in the late phase of cell injury.

Next, we investigated the involvement of Hippo signaling components in the markers of AECII-to-AECI differentiation by LPS. Our findings revealed that LPS elevated nuclear YAP levels at 6 h but decreased nuclear YAP expression while consistently increasing nuclear TAZ levels at 12 h. Previous studies have reported the activation of YAP in AECII by increasing their nuclear expression after applying mechanical tension in mice with pneumonectomy (Liu et al. 2016) and increased TAZ nuclear translocation during the late stage of AECII-to-AECI differentiation (Strunz et al. 2020; Sun et al. 2019). These findings indicate that YAP/TAZ activation is beneficial for lung epithelial regeneration and the resolution of bacterial pneumonia. However, persistent activation of YAP can lead to adverse effects, such as AECII cell senescence and impaired terminal differentiation to AECI (Chen et al. 2019; Yamada et al. 2022). Accordingly, we discovered that LPS dynamically modulated the expression of the nuclear YAP. It initially increased YAP expression in the early phase of AECII cell injury but subsequently inhibited it in the late phase. The late-phase inhibition of YAP might be beneficial in preventing cell senescence in AECII (Xu et al. 2021). In addition, the presence of nuclear TAZ in the late phase of AECII cell injury may facilitate the terminal differentiation of AECII into AECI (DiGiovanni et al. 2023).

To further clarify the role of YAP in the markers of AECII-to-AECI differentiation, we downregulated YAP in AECII. Our observations revealed a reduction in SPC and PATS markers (CLDN-4 and LGALS-3) in AECII while a slight increase in AECI marker (T1α), suggesting the progress of cell differentiating markers into AECI by YAP inhibition (Chen et al. 2023; Kondo et al. 2015; Laiman et al. 2022). Furthermore, the reduction of p53 in AECII due to YAP inhibition might lead to a reduction in cell cycle arrest associated with cell senescence (Chung et al. 2021; Gu et al. 2017), thereby promoting the late-stage of AECII-to-AECI differentiation (Kusko et al. 2016; Strunz et al. 2020). In addition, we observed a decrease in TEER in AECII at 4 h, followed by an increase at 6 h after YAP inhibition. The initial reduction in TEER indicated increased cell permeability due to AECII destruction (Metz et al. 2020). The subsequent increase in TEER could be due to the restoration of intercellular junctions among the cells, arising from an extended period of YAP inhibition and facilitating AECII-to-AECI differentiation (Ishii et al. 2021). These findings were consistent with our observations in experiments involving LPS-exposed AECII and YAP inhibition, implying that the inhibition of YAP in the late phase of AECII cell injury might stimulate AECII-to-AECI marker differentiation. Together, we suggest that LPS modulated the Hippo pathway of YAP/TAZ to progress the markers of AECII-to-AECI differentiation.

Previous studies have reported that DCLK1 is expressed in progenitor cells (Westphalen et al. 2016) and regulates cell survival and self-renewal (Chandrakesan et al. 2017). In this study, we aimed to determine DCLK1 expression in progenitor AECII and found that DCLK1 expression was decreased in isolated AECII of ARDS. These findings were consistent with our cell culture results of LPS-exposed MLE-12 at 6 h and our mouse model treated with LPS for 3 days, which showed decreased cytoplasmic levels of DCLK1 on AECII. The reduction of DCLK1 has been shown to inhibit cancer stemness and reduce the regenerative capacity of pancreatic cells (Kim et al. 2022; Westphalen et al. 2016; Weygant et al. 2014). Additionally, DCLK1 is known as a microtubule-associated protein (Agulto et al. 2021), and the process of cell differentiation depends on microtubule reorganization (Lacroix and Maddox 2014). Therefore, the transitionally reduced cytoplasmic DCLK1 observed in our ARDS models might be associated with impaired self-regenerative capacity in AECII. Notably, it has been reported that a DCLK subtype translocated to the nucleus protects kidney fibroblast-like cells from osmotic stress (Nagamine et al. 2014). In our study, we consistently observed increasing nuclear DCLK1 expression in AECII at 6 h in response to LPS. This upregulation of nuclear DCLK1 in the early phase of AECII cell injury may activate the downstream signaling pathways, promoting proinflammatory cytokine production in response to virus infection (Undi et al. 2022) and causing neutrophil activation in LPS-induced murine colitis to eliminate microbial invasion (Roy et al. 2021). However, further studies are needed to address the nuclear function of DCLK1.

Previous studies have shown that DCLK1 is involved in regulating cell survival, self-renewal, and stemness (Panneerselvam et al. 2020; Westphalen et al. 2016). It has also been reported to promote cell proliferation and activate tissue repair in the gut and pancreas (Westphalen et al. 2016; Yi et al. 2019). Given its crucial role in cellular repair, we investigated the potential involvement of DCLK1 in AECII regeneration. First, we observed increased cytoplasmic DCLK1 levels alongside an elevation in the presence of SPC^+^ cells in the lungs of ARDS mice on day 7. During the process of AECII differentiation, initial AECII proliferation is crucial to maintain an adequate AECII cell population, which can differentiate into AECI following lung injury (Bhaskaran et al. 2007; Jia et al. 2021). Therefore, the results suggest that cytoplasmic DCLK1 in the late phase of ARDS might also play a role in the AECII proliferation before their subsequent differentiation into AECI. Additionally, we found that overexpression of DCLK1 in AECII enhanced markers of differentiation into AECI by increasing T1α.

On the contrary, the knockdown of DCLK1 resulted in decreased expression of LGALS-3 and p53, suggesting that DCLK1 is essential for maintaining the regenerative capacity of AECII and facilitating their marker differentiating into AECI through the PATS cell (LGALS-3) transition. Notably, we found that DCLK1 regulates the markers of AECII differentiation through the Hippo pathway. Suppression of DCLK1 resulted in an elevation of the p-YAP/YAP ratio, whereas the overexpression of DCLK1 exhibited a non-significant reduction in this ratio. These findings suggest a potential association between DCLK1 and the Hippo-YAP pathway in AECII. Our results are consistent with previous reports of the role of DCLK1 in regulating the Hippo pathway in asthmatic AECI and airway epithelial cells through RhoA activation (Yuliani et al. 2022). These observations suggest that DCLK1 might rely on intermediary molecules, such as RhoA, to inhibit the action of large tumor suppressor kinase 1/2 (LATS1/2), resulting in YAP activation through dephosphorylation (Plouffe et al. 2016; Yuliani et al. 2022). However, the intermediary molecules linking DCLK1 and YAP in AECII of ARDS warrant further exploration in future research. Additionally, to further understand the interaction between DCLK1 and YAP, we downregulated YAP in AECII. We did not observe a significant difference in DCLK1 expression, suggesting that DCLK1 regulates YAP expression by affecting upstream components of the Hippo pathway to mediate the markers of AECII-to-AECI differentiation.

The present study provides valuable insights into the role of DCLK1 and the Hippo pathway in AECII regeneration in LPS-induced cell injury and murine ARDS. However, future research is needed to determine the long-term effects of DCLK1 on AECII differentiation and how it interacts with other cells, such as immune cells and lung fibroblasts, to promote alveolar regeneration after injury. While we identified differentiation markers in AECII, it is necessary to conduct RNA expression analysis and immunofluorescent tracking for a direct assessment of AECII-to-AECI differentiation in future works. Furthermore, additional studies are needed to explore the functions of DCLK1-regulated Hippo-YAP/TAZ pathway activation, particularly regarding dephosphorylation and nuclear translocation in ARDS. Understanding these mechanisms could help identify potential therapeutic targets for the treatment of ARDS and other lung diseases.

## Conclusions

We have shown that DCLK1 plays a crucial role in the pathophysiological repairing mechanism of AECII in LPS-induced cell injury and murine ARDS. Our study revealed decreased cytoplasmic DCLK1 expression, accompanied by increased nuclear DCLK1 and YAP levels in AECII during the early phase of ARDS. Conversely, in the late phase of ARDS, we observed increased cytoplasmic DCLK1 levels while reduced nuclear DCLK1 and YAP expression in AECII. These dynamic effects could facilitate the transition of differentiation markers from AECII to AECI. The DCLK1/Hippo pathway could be a potential therapeutic target for the treatment of ARDS in the future.

## Data Availability

Data will be made available from the corresponding author on reasonable request.
